# Case Study of a Complex Neurovascular Disorder: Choroidal Arteriovenous Malformation

**DOI:** 10.3390/medicina60020302

**Published:** 2024-02-10

**Authors:** Corneliu Toader, Razvan-Adrian Covache-Busuioc, Bogdan-Gabriel Bratu, Luca Andrei Glavan, Antonio Daniel Corlatescu, Alexandru Vlad Ciurea

**Affiliations:** 1 Department of Neurosurgery, “Carol Davila” University of Medicine and Pharmacy, 020021 Bucharest, Romania; corneliu.toader@umfcd.ro (C.T.); bogdan.bratu@stud.umfcd.ro (B.-G.B.); luca-andrei.glavan0720@stud.umfcd.ro (L.A.G.); prof.avciurea@gmail.com (A.V.C.); 2 Department of Vascular Neurosurgery, National Institute of Neurology and Neurovascular Diseases, 077160 Bucharest, Romania; 3 Neurosurgery Department, Sanador Clinical Hospital, 010991 Bucharest, Romania

**Keywords:** surgical treatment, 3D rotational DSA, 2D DSA, intraparenchymal hematoma, intranidal aneurysm, arteriovenous malformation, anterior choroidal artery

## Abstract

This study conducts an in-depth analysis of the management of a complex arteriovenous malformation (AVM) in a 44-year-old individual, who initially manifested with acute left hemiparesis and progressively declined into a comatose state. Diagnostic neuroimaging identified a substantial right fronto-temporal intraparenchymal hematoma via a CT scan. Cerebral angiography further elucidated a choroidal AVM originating from the anterior choroidal artery, accompanied by intranidal aneurysms. The elected treatment strategy was the surgical excision of the AVM. The procedure achieved complete removal of the intracranial AVM, situated in a neurologically sensitive region, leading to notable neurological recovery. This study thoroughly explores and critically evaluates a wide spectrum of treatment approaches for intracranial arteriovenous malformations, including novel endovascular therapies. Despite extensive discourse on AVM in contemporary literature, this report is among the few documenting the treatment of a choroidal AVM via a microsurgical technique, and highlights various therapeutic options.

## 1. Introduction

Arteriovenous malformations (AVMs) are defined as aberrant connections between arteries and veins, bypassing the capillary system. They exhibit epidemiological incidences ranging from 1.12 to 1.42 per 100,000 person-years, with 38–68% of cases initially presenting as hemorrhages. The annual hemorrhage rate for untreated cerebral AVMs is estimated at 2.10–4.12%. Research has concentrated on identifying determinants that increase the likelihood of AVM rupture [1]. Meta-analytical studies have correlated heightened rupture risks with factors such as previous rupture events, deep cerebral location, and exclusive deep venous drainage. Gross and Du documented an overall annual hemorrhage risk of 3.0% in observed AVMs, delineating 2.2% for unruptured and 4.5% for previously ruptured AVMs. Furthermore, Stapf et al. identified an exceptionally high annual rupture rate of 35.5% in AVMs characterized by a triad of deep venous drainage, deep cerebral location, and a history of hemorrhage [2].

Peripheral arteriovenous malformations (AVMs) represent a prevalent clinical entity, which can be systematically categorized based on the anatomical classification proposed by Shen et al. in 2023. This classification delineates four primary regions: cephalic and cervical, truncal (comprising the thoracic, abdominal, and dorsal regions), upper extremities (including the shoulder, brachium, cubital area, antebrachium, and manus), and lower extremities (consisting of the gluteal area, femoral region, genicular area, popliteal fossa, crural region, and pedal region) [3]. Furthermore, arteriovenous shunts located intracranially and within the spinal axis that drain into spinal venous structures can precipitate hypertension in the spinal perimedullary venous plexus, subsequently affecting the spinal intramedullary veins [4]. In a unique case reported by Jiang et al. in 2023, a patient suffering from a supratentorial pial AVM manifested myelopathy, characterized by notable weakness predominantly in the upper extremities, with minimal lower limb involvement, emerging two months post initial symptom onset. Post-intervention, the patient exhibited significant amelioration in cervical rigidity, and regained the ability to elevate the upper limbs and to self-dress within a week. After a period of two months, there was a near-complete resolution of the upper limb weakness, although hyperreflexia persisted in both upper and lower extremities [5].

The lack of an intervening capillary network in AVMs results in direct exposure of venous structures to elevated arterial pressures, potentially leading to serious complications such as hemorrhage. This represents an important concern in the central nervous system (CNS), where intracranial hemorrhages due to AVM ruptures can result in significant morbidity or mortality. The diagnostic and therapeutic approaches for AVMs are complex, reflecting their varied clinical manifestations and impacts on patient health. AVMs are frequently identified during evaluations following acute cerebral hemorrhage or incidentally through imaging conducted for other neurological symptoms like seizures or headaches [6,7]. As outlined by Conger et al., the diagnosis necessitates an integrated strategy encompassing patient history, physical examination, and advanced imaging techniques, including computed tomography, magnetic resonance imaging, and catheter angiography. This comprehensive approach facilitates a thorough elucidation of the AVM’s anatomical and hemodynamic characteristics.

Choroidal arteriovenous malformations, albeit infrequent, present distinct challenges due to their deep-seated location within the brain and proximity to crucial paraventricular structures [8,9]. These AVMs often exhibit recruitment of feeder vessels from both the anterior and posterior choroidal arteries [10,11].

The integration of microsurgical techniques into neurosurgical practices has facilitated the complete excision of these lesions, while preserving the integrity of adjacent critical structures. This surgical advancement has been highlighted through documented cases of successful removal of paraventricular or intraventricular AVMs [12].

In treating AVMs, the efficacy of individual therapeutic modalities is acknowledged, yet a multidisciplinary approach is frequently required to achieve optimal outcomes. Endovascular embolization, in particular, plays a versatile role as a preoperative or preradiosurgical step, and in certain cases, as a standalone curative procedure. Given that choroidal arteries traverse the lateral aspect of the ventricles, AVMs originating from these vessels pose greater challenges due to their deep location [13,14,15]. Surgical access to the anterior choroidal artery (AchA) and lateral posterior choroidal artery (LPchA) is intricate. Furthermore, AVMs situated superficially to the choroidal feeding arteries can impede surgical maneuvers. In such instances, the curative or preoperative embolization of the AchA may be advantageous, as elaborated in our research focused on endovascular AVM embolization through choroidal arteries, either as curative or adjunctive treatments [16].

We present a case involving a choroidal AVM, one of the most uncommon vascular anomalies, with limited literature available. Remarkably, our study achieved a complete resection of this AVM, leading to postoperative neurological improvement.

## 2. Case Presentation

**Figure 1:**
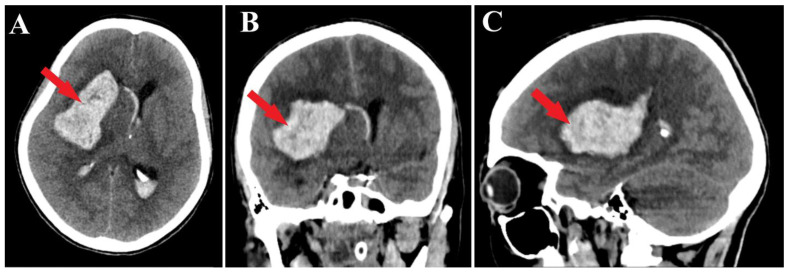
Preoperative CT scan. Axial section (**A**) highlights the intraparenchymal hematoma (red arrow); moreover, frontal (**B**) and sagittal (**C**) sections depict the massive hematoma with panventricular implication too (red arrows).

**Figure 2:**
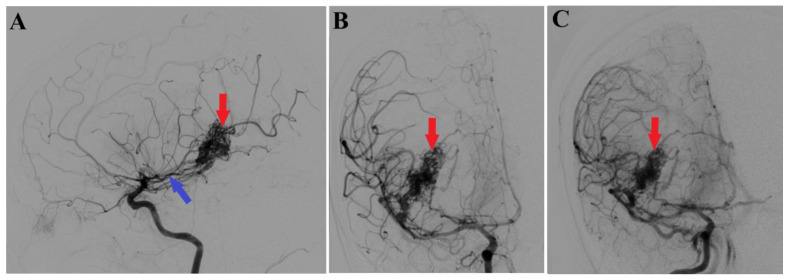
Preoperative 2D digital subtraction angiography. Profile (**A**) and frontal (**B**) 2D DSA highlights the right temporal arteriovenous malformation (red arrows), found as well in 2D DSA reconstruction (**C**). Moreover, profile 2D DSA (**A**) shows a slight dilatation of the anterior choroidal artery (blue arrow).

**Figure 3:**
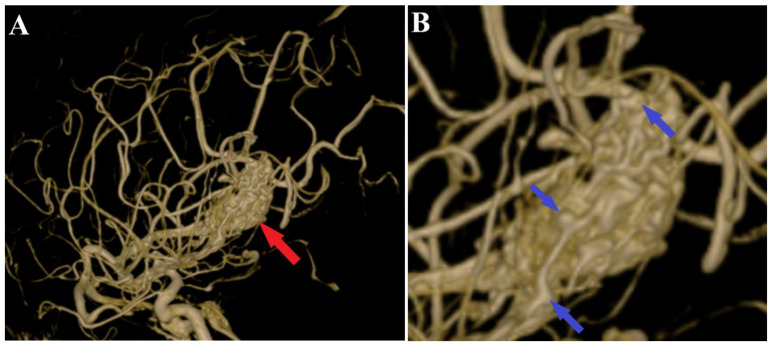
Preoperative 3D DSA rotational angiography. The 3D reconstruction of rotational DSA (**A**) depicts the tridimensional topography of the arteriovenous malformation (red arrows). In high-resolution image (**B**), multiple intranidal sacullar and fusiform aneurysms were found (blue arrows).

A 44-year-old patient was admitted to our clinic for sudden left hemiparesis MRC 1/5 occurring in the apparent health status 48 h prior to admission, followed by the deterioration of consciousness that become gradually worse, with the patient becoming comatose. She was admitted to our clinic with GCS = 7 points (eye response—2 points, verbal response—2 points, motor response—3 points), orotracheally intubated, and mechanically ventilated. Neurological examination on admission revealed a left hemiparesis, predominantly in the brachial region and comatose state. A brain non-contrast CT scan revealed a voluminous right fronto-temporal intraparenchymal hematoma with panventricular infiltration, significant mass effect on the right lateral ventricle, and 1.5 cm displacement of the midline. Otherwise, normal cerebroventricular CT appearance was seen (Figure 1). Angiography was performed through the selective injection of the internal carotid artery bilaterally, right external carotid artery, and left vertebral artery. Right temporal arteriovenous malformation with arterial afferents from the right anterior choroidal artery and right-sided middle cerebral artery was observed, Spetzler Martin grade III (Figure 2). A nidus of approximately 2/1 cm in size with several intranidal aneurysms was also observed (Figure 3). A single vein was draining into the right cavernous sinus, through an intermediate venous source.

The selected treatment approach involved the surgical removal of the arteriovenous malformation (AVM) at the right anterior choroidal artery and the evacuation of a significant deep frontotemporal intraparenchymal hematoma on the right side. Postoperatively, the patient has a favorable evolution with notable neurological recovery. The patient regained consciousness, allowing for successful extubation.

**Figure 4:**
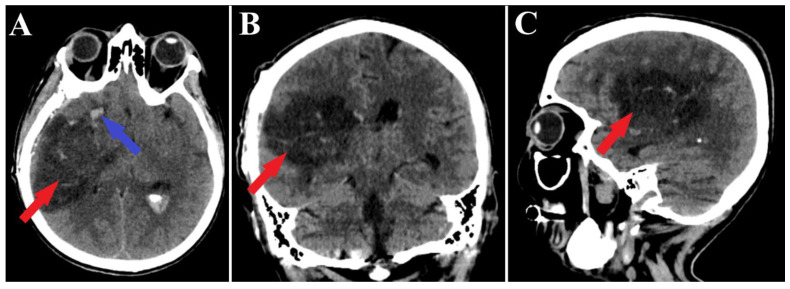
Postoperative CT scan, malformation resection shown. All three sections of the CT scan (**A**—axial section, **B**—frontal section, **C**—sagittal section) indicate a total resection of the arteriovenous malformation (red arrows), and the axial section of CT scan (**A**) depicts a small portion of an intraparenchymal hematoma (blue arrow).

**Figure 5:**
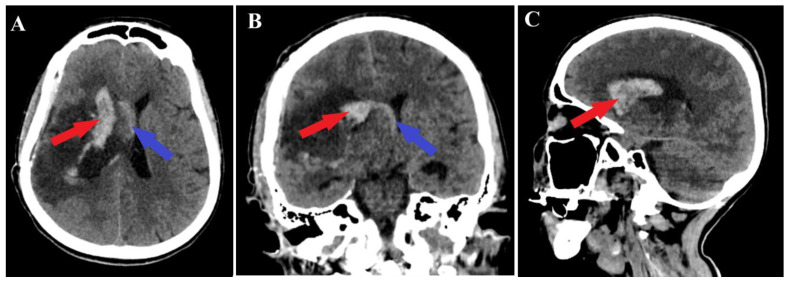
Postoperative CT scan, hematoma. Those images (**A**—axial section, **B**—frontal section, **C**—sagittal section) show a persistent pan ventricular and intraparenchymal hematoma (red arrow), as well as the contralateral intraventricular infiltration of the hematoma (blue arrows).

**Figure 6:**
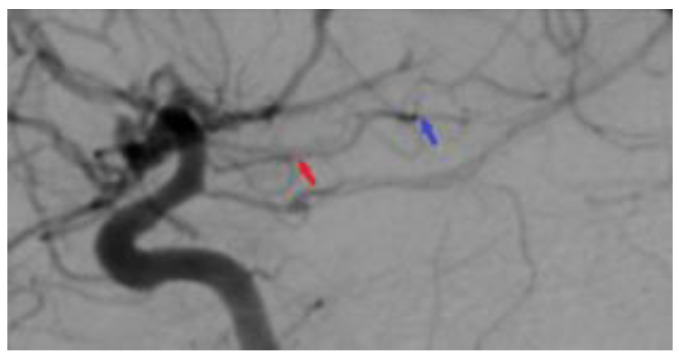
Postoperative 2D DSA. The image represents a normal diameter of the anterior choroidal artery (red arrow), with the total ablation of the right temporal arteriovenous malformation being achieved (blue arrow).

A follow-up non-contrast CT scan displayed a region of deep right fronto-temporal hypodensity, indicative of a post-surgical sequelae (Figure 4). Additionally, the CT scan identified areas of hemorrhage, which were deemed not to require further surgical intervention (Figure 5). Subsequent control angiography of the right carotid artery confirmed the complete excision of the AVM, with the preservation of the pathway of the right anterior choroidal artery (Figure 6). The patient was then managed with conservative treatment, resulting in a favorable clinical progression and substantial neurological improvement. After 2 weeks of hospitalization, at the time of discharge, the patient’s left hemiparesis had improved to 3/5 on the Medical Research Council (MRC) scale and assigned GCS was 12 points (eye response—3 points, verbal response—4 points, motor response—5 points).

## 3. Discussion

The primary aim of treating brain arteriovenous malformations is predominantly to prevent hemorrhagic events, though it may also extend to controlling seizures or halting the progression of neurological deficits [17]. Microsurgery, which involves a craniotomy and resection, is acknowledged for its low complication risk in treating small malformations located in non-eloquent brain regions, often resulting in immediate resolution. However, this approach is invasive. Stereotactic radiosurgery (SRS), a precise irradiation method, is effective for malformations under 3.5 cm, but complete eradication may take 1 to 3 years, and success is not assured. Potential delayed complications include hemorrhage and radiation-induced edema or necrosis. Embolization is utilized to manage small AVMs, prepare larger ones for (radio)surgery, or treat potential sources of hemorrhage, such as associated aneurysms. During embolization, microcatheters are used to introduce embolic materials into the feeding arteries or the nidus. Brain AVMs categorized as either Spetzler–Martin grade IV or V often necessitate a multimodal treatment approach [18]. The selection of treatment strategies is influenced by factors such as referral patterns, availability of technical resources and expertise, personal preference, health insurance policies, and a shortage of randomized controlled trials comparing different treatment modalities [19,20,21].

AVMs, situated within the cerebral ventricles, are characterized as non-eloquent in terms of their immediate anatomical positioning. However, their treatment is associated with a high-risk profile, predominantly due to the frequent presence of deep venous drainage systems. Furthermore, the surgical resection of these AVMs necessitates traversal through eloquent cortical areas, thereby elevating the complexity and potential risk of the procedure [22]. Moreover, the safe excision of ventricular AVMs presents a significant surgical challenge. This complexity is largely due to their vascular supply, which commonly includes the anterior choroidal artery (AChoA) and the posterior choroidal artery (PChoA). Additionally, the deep-seated location of these AVMs within the confines of a restricted surgical field further complicates their resection [23,24]. In the context of Gamma Knife Surgery (GKS) for ventricular arteriovenous malformations (AVMs), there have been reports of favorable obliteration outcomes, with a rate of approximately 77% achieved at a five-year follow-up period. However, it is noteworthy that the incidence of rebleeding during latency periods post-GKS for ventricular AVMs is comparatively higher than that observed in AVMs located at other anatomical sites [25].

The microsurgical excision of cerebral AVMs entails meticulous dissection to access the feeding arteries, the segmentation of the nidus and draining veins, and the minimal coagulation of the nidus. An analysis of excised AVM specimens reveals a complex vascular network with large-caliber, winding vessels, some linked to draining veins, underscoring their role in the venous aspect of the AVM. The nidus typically exhibits numerous looping formations on its surface [26].

The outcomes and postoperative complications of brain arteriovenous malformations differ markedly between patients treated with stereotactic radiosurgery and those undergoing microsurgery. Factors predictive of successful AVM obliteration post-radiosurgery include a smaller AVM size, presence of a single draining vein, lower Spetzler–Martin grade, higher margin or maximum dose, male gender, and prior history of hemorrhage [27,28]. Obliteration rates following SRS vary from 54% to 92%, with catheter angiography serving as the gold standard for diagnostic assessment. However, there is ongoing debate regarding the degree of hemorrhage protection provided by radiosurgery during the latency period, typically spanning 1–3 years. While reported hemorrhage rates during this period range from 1.6% to 9%, it is broadly acknowledged that the risk does not significantly differ from the pre-treatment period. The effectiveness of preradiosurgical embolization in enhancing SRS outcomes has shown mixed results; some studies, primarily from the pre-Onyx era, demonstrate obliteration rates of 60%-81% using this combined approach, whereas others report reduced obliteration rates associated with preradiosurgical embolization [29,30,31].

Volume-staged stereotactic radiosurgery is a potential treatment for large AVMs, known for its efficacy in controlling and obliterating these lesions. However, its application should be considered only after a meticulous, collaborative decision-making process by experienced neurosurgeons, endovascular specialists, and radio-oncologists [32]. The positioning of AVMs appears to have a negligible impact on the likelihood of eliciting radiographic alterations. However, it significantly influences the correlation between these imaging changes and the manifestation of clinical symptoms. Following radiosurgery, approximately 30% of patients exhibit new areas of high T2 signal intensity in the brain tissue surrounding the irradiated AVM nidus. These imaging changes typically emerge within a timeframe of 1 to 24 months post-radiosurgery [33].

The anterior choroidal artery (AChA) is known to supply blood to various intracranial tumors, particularly those located in the lateral ventricle, such as meningiomas, choroid papillomas [34], and gliomas [35]. Cerebral angiography often reveals these tumors to be profusely vascularized, primarily by the AChA, though additional arterial sources like the thalamoperforating or posterior choroidal arteries may also contribute [36]. Given their highly vascular nature, targeting feeders from the AChA before surgery can diminish the risk of hemorrhage and simplify surgical interventions. Consequently, preoperative embolization is advocated for to potentially reduce operative duration and is therefore recommended [37].

Arteriovenous malformations supplied by the anterior choroidal artery pose significant challenges in treatment, notably due to the elevated risk of neurological deficits post-surgery. In 1984, Fujita et al. documented the successful excision of AChA-fed AVMs, noting that factors such as the AVM’s origin from the cisternal segment of the AChA and a reduced distance between the lesion and the corticospinal tract were favorable for surgical removal [38]. The embolization of the AChA has become a recognized approach either as a precursor to surgery and radiation therapy or as an independent curative method. The goals of preoperative embolization include the obliteration of deep feeding arteries and the stabilization of AVM-associated aneurysms [39]. For instance, in 2017, Lv et al. successfully performed curative embolization in three out of four AVM cases via the AChA, with the fourth case aiding in pre-surgical preparation [16].

Nevertheless, embolization via the AChA is intricate and entails considerable risk, given that the AChA nourishes essential brain structures and lacks collateral circulation. The utilization of advanced microcatheter techniques is critical to mitigate ischemic complications. Elkordy et al., in 2017, reported enduring hemiparesis in two out of eight patients following AChA embolization for ruptured AVMs, underscoring the substantial ischemia risk [40]. To reduce these risks, some practitioners advocate for superselective provocative testing using propofol and monitoring with motor-evoked potentials. Moreover, advancements in microcatheter technology, such as the creation of smaller, flow-directed catheters, have lessened the complications associated with catheterization, thereby enabling deeper catheterization in dilated AChAs that supply AVMs [41].

Mochizuki et al. in 2023 achieved the successful embolization of AVMs supplied by the AChoA and PChoA. This was accomplished by meticulously navigating the microcatheter to a more distal and appropriate feeder vessel, a technique that remarkably resulted in the procedure being completed without any complications [13]. It is imperative to account for anatomical variations, as they can predispose to unforeseen complications during medical procedures. Moreover, as exemplified in the two cases reported in this context, particular attention should be paid to the anastomotic connections between the AChoA and PChoA, as well as between the PChoA and the anterior cerebral artery. These vascular interconnections can lead to inadvertent occlusions resulting from excessive embolization, a phenomenon that occurs due to the interconnected nature of these blood vessels [41,42].

## 4. Conclusions

In summarizing our study, arteriovenous malformations are acknowledged as intricate neurovascular entities, often accompanied by multiple complications. Concerning their overall management, endovascular therapy is extensively discussed in current literature as a comparatively safer approach for such cases. Our article features a rare instance of a choroidal AVM that was treated using microsurgical techniques, considering the eloquent localization. Additionally, our research delves into the decision-making process between endovascular and microsurgical methods, while also highlighting innovative techniques in managing this unique clinical situation.

Our case presentation sheds light on the established knowledge surrounding this pathology, including the current treatment methodologies. We also delve into the risks associated with treating this condition. The necessity for the development of novel treatment and diagnostic methods is evident, with the aim of enhancing disease control and enabling a greater proportion of patients to experience improved recovery outcomes.

## Data Availability

No new data were created or analyzed in this study. Data sharing is not applicable to this article.
